# Spatial recovery of the murine gut microbiota after antibiotics perturbation

**DOI:** 10.1128/mbio.00707-24

**Published:** 2024-06-04

**Authors:** M. Taguer, J. Xiao, R. Crawford, H. Shi, M. P. Cheng, M. Citron, G. D. Hannigan, S. H. Kasper

**Affiliations:** 1 Discovery Immunology, MRL, Merck & Co., Inc., Cambridge, Massachusetts, USA; 2 Infectious Diseases and Vaccine Research, MRL, Merck & Co., Inc., West Point, Pennsylvania, USA; 3 Informatics Technology, MRL, Merck & Co., Inc., West Point, Pennsylvania, USA; 4 Kanvas Biosciences, Inc., Monmouth Junction, New Jersey, USA; 5 Informatics Technology, MRL, Merck & Co., Inc., Cambridge, Massachusetts, USA; Rutgers, The State University of New Jersey, New Brunswick, New Jersey, USA; The University of British Columbia, Vancouver, British Columbia, Canada

**Keywords:** spatial microbiome, fluorescent in situ hybridization, multiplexed imaging, antibiotics

## Abstract

Bacterial communities are highly complex, with interaction networks dictating ecosystem function. Bacterial interactions are constrained by the spatial organization of these microbial communities, yet studying the spatial organization of microbial communities at the single-cell level has been technically challenging. Here, we use the recently developed high-phylogenetic-resolution microbiota mapping by fluorescence *in situ* hybridization technology to image the gut microbiota at the species and single-cell level. We simultaneously image 63 different bacterial species to spatially characterize the perturbation and recovery of the gut microbiota to ampicillin and vancomycin in the cecum and distal colon of mice. To decipher the biology in this complex imaging data, we developed an analytical framework to characterize the spatial changes of the gut microbiota to a perturbation. The three-tiered analytical approach includes image-level diversity, pairwise colocalization analysis, and hypothesis-driven neighborhood analysis. Through this workflow, we identify biogeographic and antibiotic-based differences in the spatial organization of the gut microbiota. We demonstrate that the cecal microbiota has increased micrometer-scale diversity than the colon at baseline and recovers better from perturbation. Also, we identify potential foundation and keystone species that have high baseline neighborhood richness and that are associated with recovery from antibiotics. Through this workflow, we add a spatial layer to the characterization of bacterial communities and progress toward a better understanding of bacterial interactions leading to improved microbiome modulation strategies.

**IMPORTANCE:**

Antibiotics have broad off-target effects on the gut microbiome. When the microbial community is unable to recover from antibiotics, it can lead to increased susceptibility to gastrointestinal infections and increased risk of immunological and metabolic diseases. In this study, we work to better understand how the gut microbiota recovers from antibiotics by employing a recent technology to image the entire bacterial community at once. Through this approach, we characterize the spatial changes in the gut microbiota after treatment with model antibiotics in both the cecum and colon of mice. We find antibiotic- and biogeographic-dependent spatial changes between bacterial species and that many of these spatial colocalizations do not recover to baseline levels even 35 days after antibiotic administration.

## INTRODUCTION

The effect of antibiotics on the gut microbiome extends beyond intended clinical outcomes, with non-target bacterial species decreasing in abundance and an overall reshaping of the gut microbiome (1–4). These disruptions to the gut microbiome lead to increased infection risk from gastrointestinal pathogens such as *Clostridioides difficile*, with extended use leading to increased risk of immunological and metabolic diseases (5–7). When prescribed early in life, antibiotic exposure is linked to an increased risk of developing asthma, allergies, and diabetes (8). While most individuals experience no detrimental effects as their microbiomes are able to functionally recover, this recovery is varied across individuals (1, 2). Recovery is influenced by many factors, from external factors, such as antibiotic class, dosage and duration, diet, and environment (9), to host factors, such as health status (10), initial microbiome composition, and diversity of resistance genes (1), to specific bacterial taxa that are associated with recovery (11). As antibiotics are a critical, life-saving drug, there is increasing interest in understanding how the gut microbiome recovers from antibiotics, and how this can be manipulated to facilitate microbiome recovery in patients taking antibiotics.

Given that recovery has been linked to the initial state of the microbiome and the presence of certain keystone species, a focus on the interactions between bacteria is warranted. Bacterial interactions are inherently a spatial question, as bacterial interactions are either physical, requiring bacteria to be in contact with one another, such as type 6 secretion systems or conjugation, or through the exchange of metabolites, where the strength of the interaction is inversely proportional to the distance between cells. Metabolic exchange between cells varies between cell type and medium, but on average occurs in small neighborhoods of a few cell lengths in scale (12).

Biogeography is also an important factor in microbiome recovery. The murine cecum has been proposed to act as a microbial reservoir after perturbations, critical for successful recovery. Cecectomized mice have lower bacterial diversity in the colon, recover slower from *Citrobacter rodentium*-induced colitis, and have increased susceptibility to infection, and when they do get infected, their recovery is slower (13, 14). Previous sequencing efforts on the luminal and mucosal communities along the length of the gastrointestinal (GI) tract in mice after antibiotics exposure found similar responses in the cecum and distal colon (15). Thus, perhaps, specific spatial changes and microbial interactions dictate the importance of the cecal microbial community in recovery from antibiotics.

To study the spatial recovery of the gut microbiota after antibiotics perturbation, we treated mice with either ampicillin or vancomycin, and analyzed the spatial relationships between bacteria at baseline, immediately after antibiotics treatment, and 5 weeks after as a recovery timepoint in both the cecum and colon. In order to visualize bacterial interactions of dozens of different bacterial taxa *in situ*, we leveraged the high-phylogenetic-resolution microbiota mapping by fluorescence *in situ* hybridization (HiPR-FISH) that utilizes advanced spectral imaging technology to label hundreds of distinct bacterial species at once and combines it with a machine learning pipeline for taxon identification (16). Through full-length 16S amplicon sequencing, we designed custom probes based on our samples to achieve amplicon sequences variant (ASV)-level resolution. To analyze spatial abundance of dozens of bacterial species and characterize changes in bacterial interactions at the micrometer scale, we developed an analytical framework for spatial microbiota diversity analysis. Spatial analysis was performed in a three-tiered approach: (i) image-level diversity to study tissue variation between the cecum and colon; (ii) pairwise colocalizations to get a global understanding of bacterial interactions and how they change in perturbation and recovery; and, lastly, (iii) focused spatial characterization of a bacterium of interest’s neighborhood that allows us to overcome the correlational limitations and inability to capture indirect interactions of pairwise analysis through a hypothesis-driven approach (17).

## RESULTS

### A mouse model of antibiotic perturbation and recovery for spatial analysis confirmed through 16S rRNA sequencing

**Fig 1:**
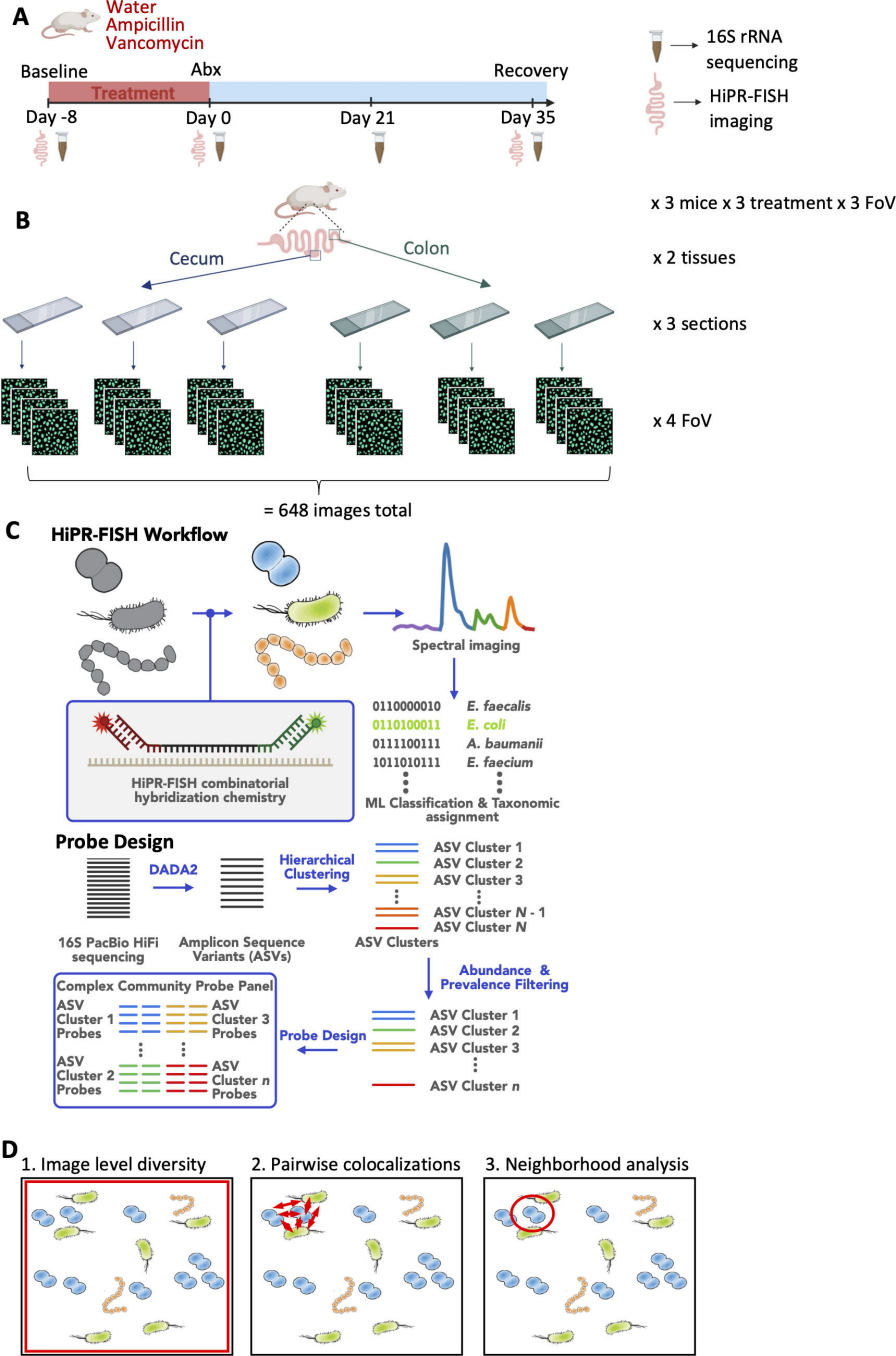
Experimental design for studying spatial recovery of the murine gut microbiota in response to antibiotics: (**A**) Mice were treated with either ampicillin or vancomycin in their drinking water for 7 days, with water-only as a control. Stool samples were collected at day −8, day 0, day 21, and day 35 for 16S rRNA sequencing. GI tissue samples were collected at day −8, day 0, and day 35 to represent baseline, treatment, and recovery timepoints for HiPR-FISH imaging. (**B**) HiPR-FISH imaging was performed on the cecum and colon of three mice per timepoint per treatment group, with three sections per tissue and four fields of view (FoV) imaged per section resulting in 36 images per tissue/treatment/timepoint and 648 images total. (**C**) HiPR-FISH combinatorial hybridization chemistry and spectral imaging enables highly multiplexed barcoding of microbial taxa. Spectra from optically barcoded microbes are measured using a confocal microscope. A machine learning algorithm classifies the spectra and assigns them to taxonomic groups. To design taxon-specific probes, full-length 16S PacBio HiFi sequencing reads are processed using DADA2 to generate ASVs. ASVs are clustered based on similarity, and probes are designed for each ASV cluster following abundance and prevalence filtering. (**D**) Spatial analysis was performed in a three-tiered approach: (1) image-level diversity; (2) pairwise colocalizations; (3) neighborhood analysis. Red squares, arrows, and circles are drawn to highlight the type and scope of the analysis performed in each method.

To characterize the spatial recovery of the gut microbiota after perturbation, BALB/c mice were given either vancomycin or ampicillin in their drinking water for 7 days, with a water-only control group. Fecal samples were collected at day −8 to establish a baseline, at day 0 immediately post the antibiotics treatment course, and at days 21 and 35 to examine recovery (Fig. 1A). Mice were monitored by measuring their water and food intake, estimated antibiotic dosage, and body weight (Fig. S1). To confirm the effects of antibiotics on the gut microbiome, qPCR and 16S rRNA sequencing were performed on the fecal samples. Bacterial loads decreased due to antibiotics and recovered by day 21, as measured through qPCR (Fig. S2A). Using 16S rRNA sequencing, we confirmed a significant decrease in alpha diversity and increase in beta diversity after antibiotics treatment that, while trending back to baseline levels, were still significantly different from baseline at day 35 (Fig. S2B through D). Despite the different mechanisms of action of vancomycin and ampicillin, with ampicillin targeting both Gram positives and negatives (18) and vancomycin targeting only Gram positives (19), we see losses of both Gram positive and negative bacteria with large-scale community remodeling (Fig. S2E). This broad impact of antibiotics on the gut microbiome has been previously observed (3, 20–23). Through established 16S rRNA sequencing methods on fecal samples, we confirmed this model of antibiotics-based microbiome disruption and continued with spatial characterization in tissue samples of microbe-microbe interactions in the recovery from antibiotics.

### HiPR-FISH-based spatial mapping of cecal and colonic microbial communities

The cecum has previously been suggested to act as a microbial reservoir, reseeding the colonic microbiome after perturbation (13). We sought to examine this hypothesis through a spatial context. To study the differences in how the cecum and colon microbiota respond to and recover from antibiotics perturbation, the HiPR-FISH method was used which allows for the highly multiplexed spatial mapping of microbial communities (16). From each tissue (*n* = 3 per treatment and timepoint), three sections were imaged with four 135 × 135 μm fields of view (FoV) (*n* = 36 images per sample group), totaling 648 images (Fig. 1B). DNA probes were designed based off full-length 16S rRNA sequences from stool samples to target unique regions of the 16S rRNA per bacterial taxonomic unit. PacBio sequencing resulted in 2.8 million reads with 97% passing denoising and chimera removal. DADA2 was used to denoise quality-filtered PacBio reads into 1,836 ASVs. The ASVs were clustered using pairwise similarity, leading to 463 ASV clusters. Probes were designed for 63 ASV clusters with an abundance of 0.2% or greater, which covered 83% of bacterial abundance, with a pan-bacterial EUB338 probe to label bacteria not captured within the probe panel (Fig. 1C) (Table S1). Spatial analysis was performed in a tiered approach, examining (i) image-level diversity, (ii) pairwise colocalizations, and (iii) neighborhood analysis (Fig. 1D).

### Improved spatial recovery in the cecum as compared to the colon

**Fig 2:**
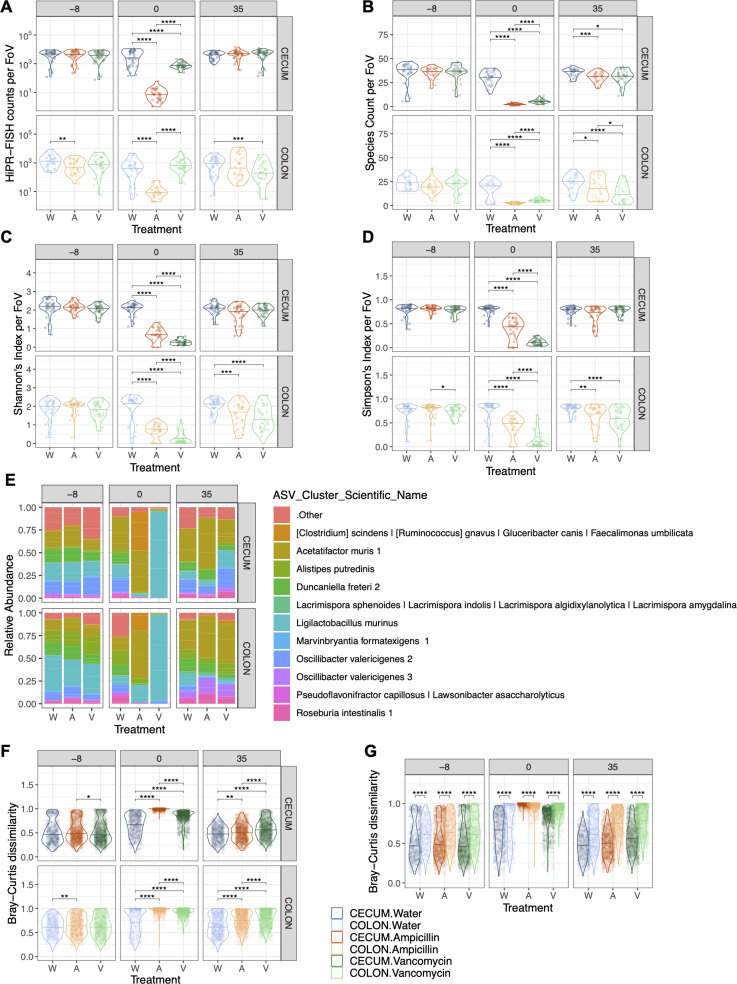
Spatial diversity after antibiotic treatment by tissue type. (**A**) HiPR-FISH counts per FoV in each treatment [water (W), ampicillin (A), vancomycin (V)], tissue, and timepoint. (**B–D**) Species count (**B**), Shannon’s diversity (**C**), and Simpson’s diversity (**D**) per FoV in each treatment, tissue, and timepoint. Statistical comparisons between treatment groups within a timepoint and tissue were analyzed with the Wilcoxon test and Holm correction for multiple comparisons. (**E**) Taxonomic diversity at the ASV level at each timepoint and treatment groups. The top 11 most abundant bacteria are plotted, with all other bacteria grouped as “Other.” (**F**) Beta diversity as measured through Bray-Curtis dissimilarity calculated for each treatment group to the water control group, within each tissue and timepoint. (**G**) Beta diversity between each treatment group and water, comparing between tissues at each timepoint. Middle lines represent median. Statistical comparisons between treatment groups within a timepoint and tissue were analyzed with the Wilcoxon test and Holm correction for multiple comparisons. *N* = 3 mice per group, 12 FoV per tissue. *P < 0.05, **P < 0.01, ***P < 0.001, ****P < 0.0001.

Alpha and beta diversity were determined based off the HiPR-FISH images to determine tissue-specific changes in response to antibiotic treatment. Both ampicillin- and vancomycin-treated mice had significant decreases in bacterial load in the cecum as compared to the water-only control group at day 0. In the colon, however, only ampicillin significantly decreased the bacterial load, and no change in bacterial load was observed in the colons of vancomycin-treated mice. In both the cecum and the colon, the largest decrease in bacterial load occurred in the ampicillin-treated mice. By day 35, the bacterial load in ampicillin-treated mice recovered to control levels in both tissues. However, while the bacterial load in the cecum of vancomycin-treated mice did recover to control levels, the bacterial load in the colon was now significantly lower than the water control group (Fig. 2A). Alpha diversity in terms of species count (Fig. 2B), Shannon’s diversity (Fig. 2C), and Simpson’s diversity (Fig. 2D) followed similar trends with large losses in diversity in the antibiotic treatment groups in comparison to the water-only group immediately following antibiotic administration. While species count did not return to control group levels in either antibiotic treatment or tissue type, Shannon’s and Simpson’s diversity did recover to control group levels at day 35 in the cecum but not in the colon. The discrepancy in the colon of vancomycin-treated mice, where there is a loss in alpha diversity but not in bacterial load, could be explained by an overgrowth of a few species in response to vancomycin treatment (Fig. 2E; Fig. S2E).

The changes in taxonomic composition depicted in Fig. 2E highlight the different responses to the two antibiotics. In the ampicillin-treated mice, the proportion of *Acetatifactor muris* and *Clostridium scindens*/*Ruminococcus gnavus* cluster represents over 75% of the community in both the cecum and colon after antibiotics, while in the vancomycin-treated mice, there is a substantial enrichment of *Ligilactobacillus murinus* in the community, representing over 95% of the bacteria found. This may explain why vancomycin did not reduce bacterial load in the colon (Fig. 2A) but did reduce alpha diversity in the same tissue at day 0 (Fig. 2B through D), as noted above. At day 35 in the ampicillin-treated mice, the *Clostridium scindens*/*Ruminococcus gnavus* cluster mostly disappears, but *A. muris* remains abundant at a higher proportion than in the water control group at the same timepoint. In the vancomycin-treated mice at day 35, *L. murinus* relative abundance decreases to levels similar to the water control group. The dominance of just one to two bacterial taxa after antibiotics exposure is striking, and the role of antibiotic resistance genes or other mechanisms of antibiotic tolerance should further be explored.

These differences in community composition between treatments (beta diversity) was next measured by Bray-Curtis dissimilarity comparing each sample to the water control group within each tissue and timepoint. The diversity between water samples demonstrates within-group variation. While there are some minor differences in beta diversity between treatment groups at baseline, these differences become much larger after antibiotics with the largest changes from ampicillin treatment. Beta diversity does not fully recover by day 35 in either treatment group and tissue type (Fig. 2F). We next compared the differences in beta diversity between tissue types and found that within sample variation is lower in the cecum than the colon when looking at baseline samples. After ampicillin treatment, the cecum is more perturbed than the colon, and yet the cecum recovers better than the colon after either antibiotic by day 35 (Fig. 2G). To note, while there is some variation in both alpha and beta diversity across the baseline days, we attribute this mostly to cage effects due to the large amount of variation that can exist across gut microbiomes. These differences tend to be smaller than the differences seen due to treatment or tissue.

In summary, the acute effects of both antibiotics similarly disrupt the communities of the cecum and colon, while the cecum has a higher capacity for recovery than the colon with more measures of diversity reaching water control levels at day 35 compared to the colon.

### Baseline diversity at the microscale is higher in the cecum than the colon

**Fig 3:**
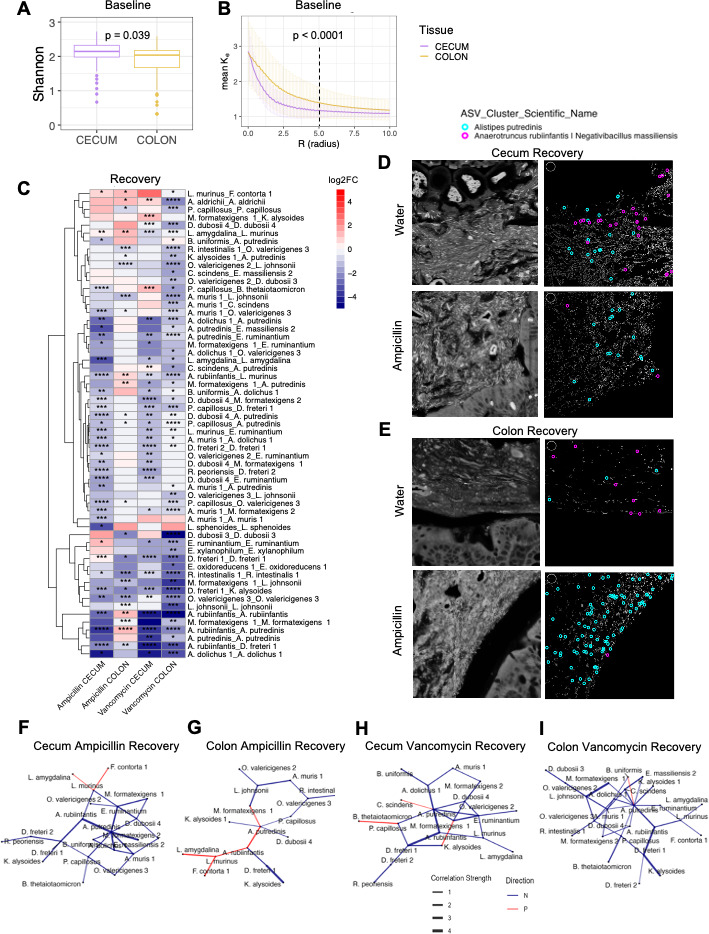
Pairwise diversity in the cecum and colon at baseline and in recovery. (**A**) Alpha diversity, as measured by the Shannon index, at the image level at baseline on all samples at day −8. (**B**) Alpha diversity, as measured by a nearest neighbor analysis, for every pairwise colocalization on all samples at day −8. (**C**) Heatmap of the log2 fold change in colocalization scores between treatment groups and water-only control at day 35. Pairs of bacteria that have a significantly different colocalization score between antibiotic and control group in at least one tissue and treatment group after prevalence, abundance, and log2FC filtering were used to build this heatmap of the log2FC of the colocalization scores. Filtering thresholds were set to exclude bacteria found less than 2,000 times across the study and in less than 25% of images or with small log2FC between −1 and 1. Treatment and tissue groups are on the *x*-axis, and pairwise colocalizations on the *y*-axis, with the format Bacteria1_Bacteria2. Bacterial pairs that had an increased colocalization score at day 35 in the treatment group compared to the water group at day 35 have a positive log2FC, whereas bacterial pairs that had a decreased colocalization score in the treatment group compared to water have a negative log2FC. *P < 0.05, **P < 0.01, ***P < 0.001, ****P < 0.0001 (Kruskal-Wallis test with Benjamini-Hochberg adjustment, *n* = 6 mice per group, 12 FoV per tissue). (**D, E**) Representative images of the colocalization between *Alistipes putredinis* and *Anaerotruncus rubiinfantis* in the cecum (**D**) and colon (**E**) at day 35 with and without ampicillin treatment. Spectral max projections on the left and segmented images with the two bacteria encircled on the right. A white circle with a 5 µm radius is included in a top corner for scale. (**F, G**) Network of changes of bacterial interactions at a 5 µm radius in the cecum (**F**) and colon (**G**) of ampicillin-treated mice at day 35 compared to control. (**H, I**) Network interactions in the cecum (**H**) and colon (**I**) of vancomycin-treated mice. Only significant interactions are included, self-interactions are removed. Line thickness depicts strength of the change and color depicts directionality (red, more interactions at recovery; blue, fewer interactions at recovery).

We next hypothesized that the cecum’s improved recovery over the colon after antibiotics exposure could be related to increased alpha diversity at baseline, as alpha diversity is typically a hallmark of resilient microbial communities and associated with health (24). At the image level, the cecum is slightly (12%) more diverse than the colon at baseline (Fig. 3A). We decided to investigate this small but significant difference further by looking at the alpha diversity at a micrometer-level resolution. To do this, we used the nearest neighbor approach for all samples collected at day −8 before antibiotics were administered. The nearest neighbor approach used here is where for each bacterium, the conditional probability of the nearest bacterium being of the same species or different species was estimated for every distance in radius (r, from 0 to 10 µm). This is calculated using the mark correlation function kf(r). Values <1 indicate that at a distance (r), there is a lower than expected probability that the two bacteria are of the same species, whereas values >1 have a higher than expected probability that the two bacteria are of the same species. At smaller distances of around 5 µm or less, both the cecum and colon have values >1, suggesting that bacteria of the same species tend to colocalize together (Fig. 3B). This could be due to a lack of mobility after replication and cell division, and represents increased cell proliferation. However, the probability of the nearest neighbor being of the same species is lower in the cecum than the colon at these small distances (Fig. 3B), and so at the micrometer level, the cecum is more heterogeneously dispersed than the colon with increased diversity of potential microbe-microbe interactions. Above 5 µm, the probability approaches 1, indicating that at larger distances, the colocalization effect of bacterial species is lost. At distances greater than 5 µm, whether two bacteria are of the same species or not becomes a stochastic process. We postulate that the cecum’s increased microscale diversity as compared to the colon could be partially responsible for the increased resilience seen in the cecal microbial community as compared to the colon’s microbial community.

### Changes in pairwise colocalizations differ by tissue and antibiotic

We next focused on characterizing the pairwise colocalizations that occur within a 5 µm radius. To do this, every pair of bacteria within 5 µm of each other was counted and compared to the probability of those two bacteria occurring together given their abundance in the image. This results in a colocalization score for each bacterial pair, where a value >1 indicates that the colocalization occurs more often than expected, and a score <1 indicates that the colocalization occurs less often than would be expected if bacterial interactions were random.

We then analyzed each pairwise colocalization to determine how much its colocalization score changed due to antibiotic treatment by comparing each treatment group to the water-only control group at each timepoint and in each tissue (Table 1). Because the effect was so large immediately post-antibiotics due to large decreases in bacterial load at day 0, we focused on the recovery timepoint to further understand and characterize recovery through a spatial lens. We plotted the difference in colocalization scores of the pairwise colocalizations between the antibiotic-treated mice and the water-only control mice at the recovery timepoint day 35 for each tissue (Fig. 3C). We filtered the 4,624 possible pairwise colocalizations based on abundance, prevalence, log2 fold change, and significance, which yielded 59 unique pairwise colocalizations that were significantly different and had a log2FC greater than 1 or less than −1 between either antibiotic treatment or water control in either the cecum or colon (Fig. 3C). For each of the 59 pairs of bacteria, the colocalization scores were plotted across all tissues and treatments. The majority of the changes in colocalization scores occurred in the colon of vancomycin-treated mice with 50 significant changes in score, compared to 35 changes in the cecum of these mice. This further highlights effects of different antibiotics on specific bacterial interactions, and how this differs according to biogeography (Fig. 3C).

**TABLE 1:** Summary of the changes in pairwise colocalizations after antibiotics perturbation and recovery^*a*^

Tissue	Treatment	Day	Signif	Large_effectsize	TotalColocs	% Signif	% Large_EffSize
Cecum	Ampicillin	−8	42	19	1,250	3.36	1.52
Cecum	Vancomycin	−8	5	5	1,258	0.40	0.40
Cecum	Ampicillin	0	580	398	825	70.30	48.24
Cecum	Vancomycin	0	579	413	833	69.51	49.58
Cecum	Ampicillin	35	214	104	1,038	20.62	10.02
Cecum	Vancomycin	35	287	129	1,107	25.93	11.65
Colon	Ampicillin	−8	1	1	668	0.15	0.15
Colon	Ampicillin	0	260	172	458	56.77	37.55
Colon	Vancomycin	0	282	160	478	59.00	33.47
Colon	Ampicillin	35	87	32	736	11.82	4.35
Colon	Vancomycin	35	225	87	712	31.60	12.22

^*a*^ Pairwise colocalization scores were compared to the water-only control group at each timepoint and each tissue using a pairwise Kruskal-Wallis test and Benjamini-Hochberg correction for multiple comparisons. The Kruskal-Wallis effect size was calculated on pairwise colocalizations that were significantly different from baseline at a *P*-value of 0.1, and categorized as “large” if the effect size (EffSize) was equal or greater than 0.14. A list of each significant pairwise colocalization is in Table S2.

To further visualize these differences in pairwise colocalizations, we chose a bacterial pair with a colocalization score that differed depending on the biogeographic location. This pair is *Alistipes putredinis* and the cluster *Anaerotruncus rubiinfantis | Negativibacillus massiliensis*. To analyze these images, spectral max projections were used for segmentation into individual bacterial cells. We then overlaid circles highlighting these two bacterial species in the segmented image to visualize the decrease in colocalization score in the cecum (Fig. 3D) and increase in the colon (Fig. 3E) after ampicillin treatment. Even with the low abundance of *A. rubiinfantis* in the cecum after ampicillin treatment, it was consistently colocalized to multiple *A. putredinis* bacteria more so than what would be expected by chance, leading to a high colocalization score (Fig. 3E). To visualize the entire network of bacterial interactions within a 5 µm radius, we built an association network from the colocalization scores used to generate the heatmap for both tissues and antibiotics (Fig. 3F through I).

### Neighborhood analysis identifies specific changes in bacterial environments

To move beyond looking at pairwise colocalizations, we applied a neighborhood-centric approach to each bacterium systematically. For every bacterium, all other bacteria found within its 5 µm radius were listed to create the neighborhood diversity for each “center bug.” This enabled us to quantify and characterize the changes in each bacterium’s neighborhood in response to antibiotics perturbation, and the biogeographical dependence of those changes. We approach this by calculating the permutational multivariate analysis of variance (PERMANOVA) for every bacterium’s neighborhood between the treatment group and the water-only control at day 35 to see which ones changed significantly and identified the bacterium within the neighborhood driving these changes. A list of every bacterium’s neighborhood that changed significantly (p < 0.1), along with the bacterium with the largest coefficient, is listed in Table S2.

**Fig 4:**
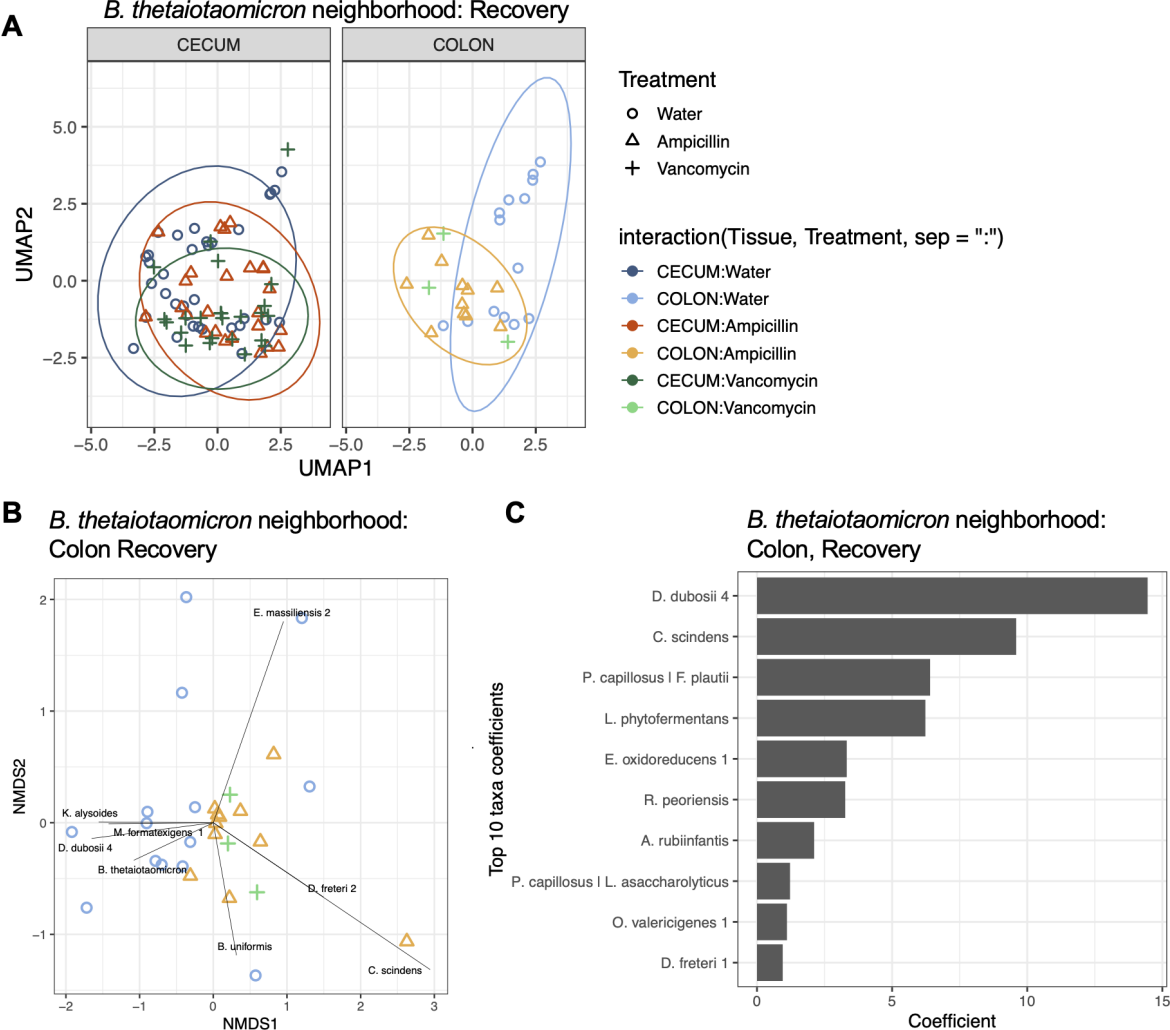
Changes in the spatial neighborhood of *B. thetaiotaomicron* due to antibiotics treatment. (**A**) UMAP of the recovery of *B. thetaiotaomicron*’s neighborhood (radius = 5 µm) by treatment per tissue by comparing treatment to water control at day 35 (shape: open circle = water, open triangle = ampicillin, plus sign = vancomycin). (**B**) NMDS biplot of colon samples at day 35. Arrows represent specific taxa driving the differences in clustering of samples. The length of the vector represents effect size, and direction represents increased influence on cluster differentiation. (**C**) The PERMANOVA coefficients of the top 10 bacteria associated with the difference between antibiotics for recovery in the colon of ampicillin-treated mice. *N* = 3 mice per group, 12 FoV per tissue.

To provide an example of this analytical workflow, we characterized the changes in *Bacteroides thetaiotaomicron*’s neighborhoods over the course of antibiotic treatment. *Bacteroides thetaiotaomicron* was selected as it had the largest significant change in neighborhood diversity due to antibiotic treatment at day 35, which was in the colon (Table S2). For every instance of *B. thetaiotaomicron*, all the bacteria found within the 5 µm radius are identified. This leads to a multivariate data set that can be analyzed and visualized with typical ordination techniques. Uniform manifold approximation and projection (UMAP) provides an overview of the neighborhood composition for both the cecum and colon at recovery for all three treatment groups, showing the different clustering of ampicillin and vancomycin from the water control in the colon but not in the cecum (Fig. 4A). We next focused in on the colon to further characterize the changes in *B. thetaiotaomicron*’s neighborhood. A non-metric multidimensional scaling (NMDS) biplot, where the points represent samples and the arrows represent the eigenvalue coefficients and vectors, reveals which bacteria are most responsible for the separation between treatment groups. *Duncaniella dubosii 4*, *Marvinbryantia formatexigens,* and *Kineothrix alysoides* drive the separation of both ampicillin- and vancomycin-treated mice away from the water-only controls (Fig. 4B). Similarly, the coefficients behind the PERMANOVA can be used to assess the differences driving changes in *B. thetaiotaomicron*’s neighborhood between different conditions (Fig. 4C). This orthogonal method identifies many of the same bacteria driving the separation between treatment groups. As these pairwise colocalizations were not depicted in the heatmap in Fig. 3, this bottom-up, hypothesis-driven analysis allows us to further characterize changes in microbial neighborhoods that are lost when only looking at pairwise comparisons.

### Keystone and foundation bacteria correlate with recovery post-antibiotics

Keystone species are bacteria that, while low in abundance, play important roles in community stability and recovery from perturbation. In contrast, foundation species are more abundant members of a community which are also important for driving community structure. Both types of members play critical roles in community stability and recovery due to their interaction networks and ability to modulate the local environment toward favorable conditions allowing later stage colonizers to grow (25, 26).

We hypothesized we could identify bacteria that potentially meet the definition of keystone and foundation species based on their neighborhood richness. Bacteria with high neighborhood richness—the number of unique species found within a bacterium’s 5 µm neighborhood—have increased opportunity for interbacterial interactions and cross-feeding events. These bacteria would be important for ecosystem recovery, and so we next hypothesized that their abundance at baseline would correlate with community recovery.

**Fig 5:**
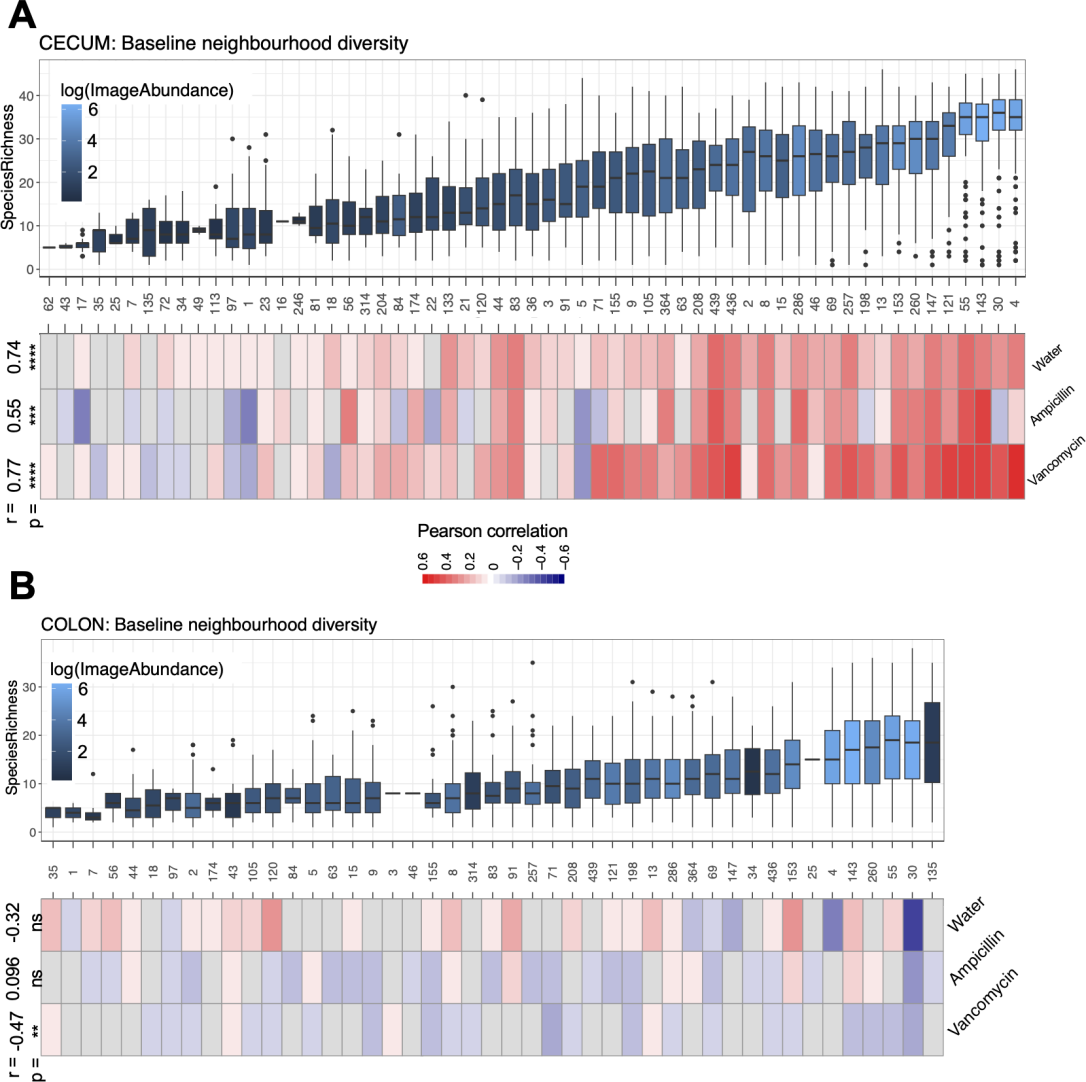
Neighborhood diversity is correlated with community recovery. Neighborhood richness, as defined by the number of unique bacterial species within a 5 µm radius of every bacterium across baseline images, is plotted for the (**A**) cecum and (**B**) colon. Numbers on the *x*-axis represent bacteria by their bacterial IDs which are listed in Table S1. Boxplots are ordered by mean species richness, while boxplots represent the interquartile range with the middle line representing the median. Boxplots are color-coded by the log mean abundance of the bacterium at baseline. Heatmaps representing the correlation between abundance of the bacterium at baseline versus recovery of samples calculated as 1 − Bray-Curtis between every day −8 sample and every day 35 sample. Only significant correlations are included. The Pearson correlation coefficient and *P*-value of the trend between the bacterium’s neighborhood richness and importance in recovery are written at the top of the heatmap on the left. *N* = 3 mice per group, 12 FoV per tissue. *P < 0.05, **P < 0.01, ***P < 0.001, ****P < 0.0001.

To look for keystone and foundation species in these data, we first calculated neighborhood richness for each bacterium at baseline (day −8). Then, to differentiate between low abundance and high abundance bacteria and thus between keystone and foundation species, we colored the boxplots based on the average abundance of each “center bug” (Fig. 5A). Then, for each “center bug,” we calculated the correlation between abundance at baseline and community recovery. Community recovery was measured as the beta diversity at the image level between recovery and baseline. There is a clear and significant correlation between a bacterium’s neighborhood richness and its importance in community recovery in the cecum (Fig. 5A) but not the colon (Fig. 5B). This correlation varied between treatments, with the strongest correlation in the vancomycin-treated mice, and no correlation in the colon of the ampicillin- and water-treated mice. An example of a correlation plotted in the heatmap is in Fig. S3. Lastly of note, the similarity between two timepoints before and after a perturbation is a measure of recovery, whereas when there is no perturbation, it is a measure of stability. Thus, in the water control group we are measuring community stability.

With this method, we identified potential keystone species due to their high neighborhood richness, correlation with recovery, and low abundance. The one bacterium with high neighborhood richness (top 25th percentile), low overall abundance (bottom 80th percentile), and strong correlations to recovery (top 25th percentile) is *Bacteroides uniformis* (ID #198). The potential foundation species we identified through this method, having high abundance and the highest neighborhood richness, are *Oscillibacter valericigenes 2*, *L. murinus*, *A. muris*, and *Duncaniella freteri 2*.

## DISCUSSION

In this study, we applied a highly multiplexed imaging method, HiPR-FISH, to study the spatial perturbation and recovery of the gut microbiota after antibiotics. To analyze such data, we developed an analytical framework that involved three major analysis pipelines: (i) image-level diversity, (ii) pairwise colocalizations, and (iii) neighborhood analysis. Through this framework, we describe large disturbances in the microbial communities after antibiotics that mostly recovered by day 35 but still see substantial changes at the micrometer scale between pairwise colocalizations. We also described a neighborhood approach that extends bond pairwise analysis and demonstrated how this approach could be used to identify foundation and keystone species that are seemingly important for community recovery through their high neighborhood richness and correlation with community recovery.

Through this method, we identified *B. uniformis* as a potential keystone species. *Bacteroides* species tend to have large repertoires of carbohydrate-degrading enzymes, commonly acting as primary colonizers producing metabolites for secondary colonizers (11, 27). Specifically, *B. uniformis* has previously been identified as a bacterium associated with recovery from antibiotics from a meta-analysis of four human cohorts (11). We also identified *O. valericigenes 2*, *L. murinus*, *A. muris*, and *D. freteri 2* as potential foundational species. *O. valericigenes* and *A. muris* are capable of high levels of short-chain fatty acid production (28), *L. murinus* is an abundant lactic acid-producing bacterium (29), and *D. freteri* is a known mucus degrader (30). These are all important metabolic processes in the gut; however, the presence of Firmicutes rather than mostly Bacteroidetes in this group is unexpected. Perhaps, the more specialist nature of Firmicutes metabolism (31) requires them to be more reliant on these cross-feeding interactions and thus are most commonly found in neighborhoods with high richness. The biogeographic differences in diversity and recovery have been previously seen with similar spatial approaches such as MaPs-Seq (32). 32

Highly multiplexed imaging platforms like HiPR-FISH and others (33–35) are allowing for untangling the complexities of spatial organization at single-cell resolution of complex microbial communities. Understanding how the spatial organization between bacteria change over time and in response to perturbation allows us to better understand bacterial organizations and co-dependency networks that underpin bacterial metabolism. FISH-based imaging approaches have two main benefits over standard sequencing-based approaches: firstly, they allow for measurements in absolute abundances, overcoming the limitations imposed in compositional data and working with relative abundances that are necessitated by current sequencing approaches (36). Secondly, rRNA hybridization ensures that only bacteria with moderately high levels of rRNA are included, precluding exogenous DNA or nonviable bacteria from being incorporated into count data. This differs from standard 16S sequencing approaches where exogenous DNA and DNA from dead bacteria may get captured, along with the biases associated with amplification and sequencing in analysis (37).

While imaging is a markedly different approach from sequencing for diversity analysis, it has its own unique experimental and analytical limitations. Experimentally assessing probe specificity in complex samples is challenging, and so experimental specificity is difficult to confirm. Additionally, probe penetration and hybridization to rRNA is limited to certain cellular physiologies, with differing fluorescence intensity depending on growth phase and rRNA content (38–40). As suggested above, this can be viewed as a benefit or limitation. By designing custom sample-specific probes based off full-length 16S rRNA gene sequences, off-target hybridization is computationally avoided and differentiation between phylogenetically similar species is improved upon compared to using short-read sequencing. Analytical limitations in multiplexed 16S rRNA FISH methods include the challenges in cell segmentation of dense and complex microbial communitites and, perhaps specific to this method, analysis is based off cell centra and so differences in cell size are not incorporated into analysis.

Highly multiplexed imaging of microbial communities enables the spatial analysis of complex bacterial associations and potential interactions. In this work, we present an analytical framework for analyzing these new data sets that can be applied to many different experimental designs. An exciting path forward in multiplexed imaging is to combine methods like HiPR-FISH with host labeling, such as through spatial transcriptomics or multiplexed immunofluorescence to obtain insight on microbiome-host interactions. Such interactions and how they change after a perturbation can lead to new hypotheses of mechanisms of action and further help resolve the field of microbiome-host interactions.

## MATERIALS AND METHODS

### Mice and antibiotics treatment

Female 8-week-old specific pathogen-free BALB/c mice were purchased from Taconic Farm. Mice were acclimated for 2 weeks before the start of the experiment. Mice were housed six per cage, with three cages per treatment. Mice were treated with 1 mg/mL ampicillin or 0.5 mg/mL of vancomycin in their drinking water for 7 days. Stool samples from each mouse were collected at baseline (day −8), immediately after the antibiotics course (day 0), at day 21, and at day 35, and stored in 70% ethanol at −20°C until DNA extraction. Two mice per cage were sacrificed at day −8, day 0, and day 35, and gut tissue was harvested and fixed in Carnoy’s solution (60% ethanol, 30% chloroform, and 10% glacial acetic acid) for 48 h at room temperature, rinsed three times in 70% ethanol, and stored in 70% ethanol at −20°C until sectioning and imaging. Three mice per timepoint and treatment group were sent for HiPR-FISH imaging.

### DNA extraction, 16S rRNA gene sequencing, and analysis

DNA extractions and sequencing were performed by CosmosID. DNA was extracted from mouse fecal pellets using the Qiagen PowerSoil Pro Kit according to manufacturer’s protocol. Sequencing protocols were performed as described in reference 41. Sequences were filtered based off control samples (MSA 2002 from ATCC); operational taxonomic units (OTUs) with a count less than 20 or that were found in less than 1% of samples were filtered out. Samples with a sequencing depth less than 3,000 were removed. Alpha diversity was calculated using the Shannon index, and beta diversity was calculated using the Bray-Curtis dissimilarity index with the vegan package (v.2.6–4) and the rstatix package (v.0.7.1)

16S rRNA qPCR was performed on the extracted DNA to determine 16S rRNA gene copy number as a proxy for bacterial load with the Zymo Femto Bacterial Quantification Kit (Cat #E2006). Standard curve was generated with a serial dilution of *Escherichia coli* strain JM109 and ZymoBIOMICS microbial standard (Cat #D6300) as the positive control. Variation between controls on different qPCR plates led to standardizing cell count data to baseline and plotting as the fold change from baseline.

### HiPR-FISH probe design and imaging

Kanvas Biosciences completed FISH probe creation, sample processing, imaging, and image preprocessing as part of their HiPR-Map service.

#### Probe design

PacBio sequencing was performed on pooled fecal samples from baseline. FASTA sequences from full-length 16S PacBio sequencing were grouped in similar taxa by sequence similarity of ASVs. In total, 463 ASV groups were identified. Among these ASV groups, 63 groups have relative abundances of at least 0.2% across all sequencing data sets and were selected for probe design. For each of the 63 selected ASV groups, different probe sequences with high specificity were selected and concatenated with landing pads corresponding to secondary fluorescent readout oligos. When multiple ASV groups belonged to the same species, numbers were added sequentially to the end of the species name to distinguish between different 16S rRNA sequences for the same species.

#### Sample processing

For each specimen, the colonic portion of the GI tract was dissected into roughly 1 cm-length blocks. Whenever possible, the tissue was cut at places where there were no fecal pellets to keep pellet and digesta region intact for imaging. Cecum was dissected away from the small intestine and large intestine. Dissected samples were paraffin embedded and sectioned to 4 µm by the Histology Laboratory at Animal Health Diagnostic Center, College of Veterinary Medicine, Cornell University. Sections were stored at 4°C until hybridization.

#### Hybridization

For deparaffinization, tissue sections on glass slides were incubated at 60°C for 10 min, washed once in xylene substitute solution (VWR, Cat #89370-090) for 10 min, once in xylene substitute solution at room temperature for 10 min, and once in ethanol at room temperature for 5 min, and air dried. To reduce autofluorescence, deparaffinized slides were washed with 1% sodium borohydride in 1× phosphate-buffered saline (PBS) on ice for 30 min, with a buffer change every 10 min, followed by three washes in 1× PBS on ice for 5 min each. Slides were briefly dipped in ethanol and allowed to air dry. Tissue samples were permeated by deposition of 50 µL of 10 mg/mL lysozyme suspended in 10 mM Tris-HCl onto the slide and incubation at 37°C for 30 min. After lysozyme digestion, the coverslips were removed by depositing an excess of 2× saline-sodium citrate (SSC). Slides were then washed in 1× PBS for 15 min, dipped in pure ethanol, briefly rinsed with pure ethanol to remove any residual PBS, and air dried. For encoding hybridization, 50 µL of encoding hybridization buffer was deposited on the tissue and a glass coverslip was placed over the tissue to ensure even distribution of the hybridization buffer. Encoding hybridization was performed at 37°C for 18 h. The slides were then washed in the washing buffer at 48°C for 15 min, dipped in room temperature absolute ethanol, rinsed with absolute ethanol, and air dried. Readout hybridizations were carried out similarly (50 µL readout hybridization buffer per slide) at room temperature for 2 h. The slides were washed and dried as described above and embedded in 20 µL ProLong Gold Antifade embedding medium and allowed to cure overnight before imaging.

#### Imaging and image processing

Specimens were imaged on a Zeiss i880 confocal in spectral mode. For each specimen, Kanvas Biosciences collected four fields of view, each with a size of 135 µm × 135 µm. Spectral data were collected using multiple laser excitations between 405 nm and 633 nm generating emission spectra between 405 nm and 680 nm. Images were processed using Kanvas’ proprietary software. Briefly, each microbe was segmented to determine cell boundaries. The spectra within the boundaries of each segmented object were compared to Kanvas’ database to perform barcode identification and provide quality metrics.

### HiPR-FISH spatial analysis

#### Image-level diversity analysis

Bacterial counts per image (FoV) were used for the alpha and beta diversity analysis, using the Shannon and Bray-Curtis dissimilarity indexes, respectively. Pairwise Wilcoxon test with Benjamini-Hochberg (BH) correction for multiple comparisons was used to compare between tissue types. Log count data were used for the UMAP ordination (neighborhoods = 10) and PERMANOVA (permutations = 999) for the statistical analysis between timepoints within treatment and tissue groupings.

#### Pairwise colocalization analysis

The colocalization score was generated by dividing the proportion of each pairwise colocalization in a sample by the probability of those two bacteria occurring together given their relative abundance in the image. This led to a probability scoring system, with values >1 indicating the pairwise colocalization occurred more often than expected if the events were independent, and values <1 indicating the pairwise colocalization occurred less often than expected if the events were independent. UMAP ordination was performed with neighborhoods = 10, and PERMANOVA with 999 permutations. Statistical analysis to determine the changes in the pairwise colocalization scores over time was performed with the Kruskal-Wallis test with BH adjustment for multiple comparisons.


CS=P(AB)÷P(A)P(B)

CS is the colocalization score and $P\left(AB\right)$ is the proportion of bacteria A and B colocalized together. $P\left(A\right)$ is the proportion of bacteria A and P(B) is the proportion of bacteria B.

#### Nearest neighbor analysis

To determine if the nearest neighbor to any given bacterium is of similar type or different type, we used the markcorr function from the spatstat package (42) which uses a special case of the mark correlation function called the standardized mark equality function. It outputs the conditional probability that two points are of the same type given any radius. We worked within the range of 0–10 μm.

#### Neighborhood spatial analysis

A count matrix is generated where for every “center bug,” the count of each “neighbor bug” within a 5 µm radius is generated. This matrix is then divided by the expected count of that neighbor bug in a neighborhood. The expected count is calculated by dividing the proportion of the “neighbor bug” in a sample by the total number of bacteria found within “center bug’s” neighborhood. This colocalization score is interpreted the same way as the pairwise colocalization score (i.e., values >1 occurred more often than expected and values <1 occurred less often than expected). Count matrixes were generated in Python and statistical analysis was performed in R (v.4.1.1) with the vegan package (v.2.6–4).


$$N_{y\;}^{x}=\;x_{y}^{c}÷\left(x^{p}\times n_{y}^{c}\right)$$

$N_{y\;}^{x}$ is the neighborhood colocalization score of bug x in bug y’s neighborhood. $x_{y\;}^{c}$ is the count of bug x in bug y’s neighborhood, **$x^{p}$** is the total proportion of bug x, and is the total count of bugs in bug y’s neighborhood.

## Data Availability

Data and analysis code are available at Github (see https://github.com/MSDLLCpapers/mtag-Spatial_Abx_Manuscript) and at figshare (see https://figshare.com/articles/dataset/Spatial_recovery_of_the_murine_gut_microbiota_after_antibiotics_perturbation_-_Raw_data/25766280).

## SUPPLEMENTAL MATERIAL

Supplemental Figures mbio.00707-24-s0001.pdf Figures S1-S3.

Supplemental Legends mbio.00707-24-s0002.docx Legends for supplemental tables and figures.

Table S1 mbio.00707-24-s0003.csv List of bacterial clusters identified by HiPR-FISH probes.

Table S2 mbio.00707-24-s0004.csv List of bacteria with significantly different neighborhoods in recovery after antibiotic treatment.

ASM does not own the copyrights to Supplemental Material that may be linked to, or accessed through, an article. The authors have granted ASM a non-exclusive, world-wide license to publish the Supplemental Material files. Please contact the corresponding author directly for reuse.
