# Supplementary material for: Spatial recovery of the murine gut microbiota after antibiotics perturbation
Source: mBio. 2024 Jun 4;15(7):e00707-24. doi: 10.1128/mbio.00707-24 (PMC11253616; doi:10.1128/mbio.00707-24)
Supplement: Supplemental Figures — Figures S1-S3. [file mbio.00707-24-s0001.pdf]

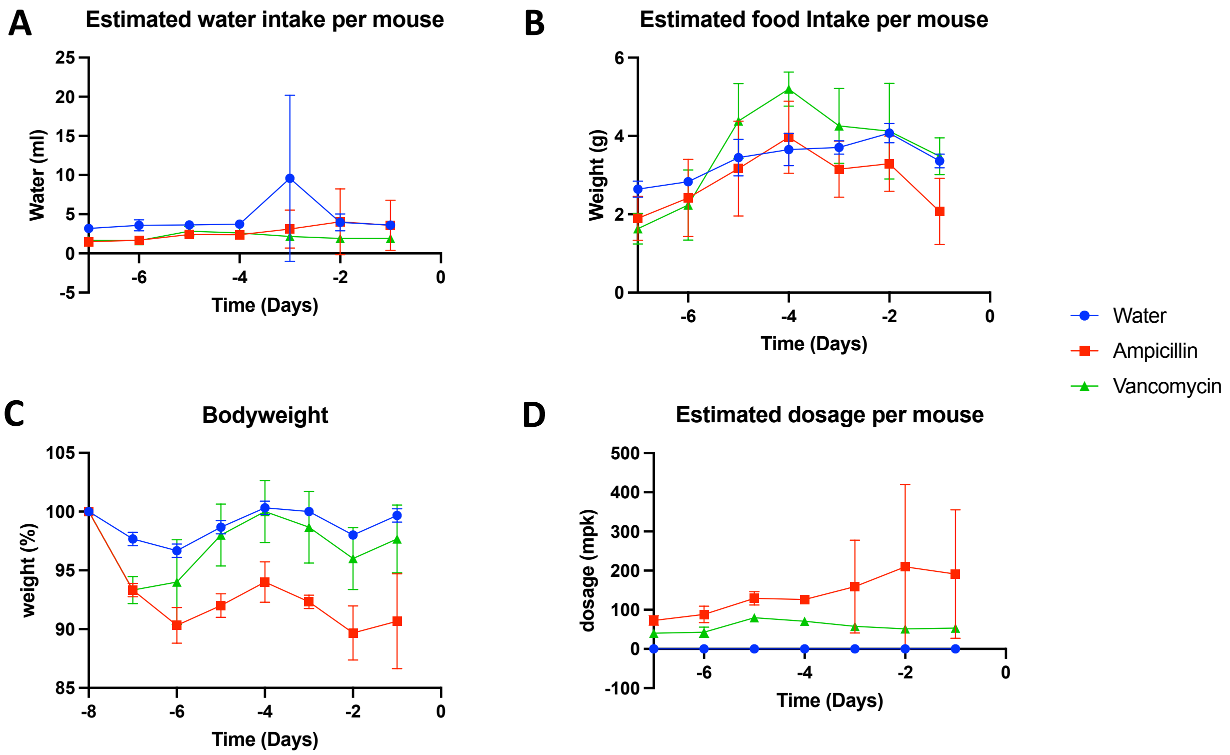
A
Estimated water intake per mouse
Water (ml)
Time (Days)
B
Estimated food Intake per mouse
Weight (g)
Time (Days)
Water
Ampicillin
Vancomycin
C
Bodyweight
weight (%)
Time (Days)
D
Estimated dosage per mouse
dosage (mpk)
Time (Days)

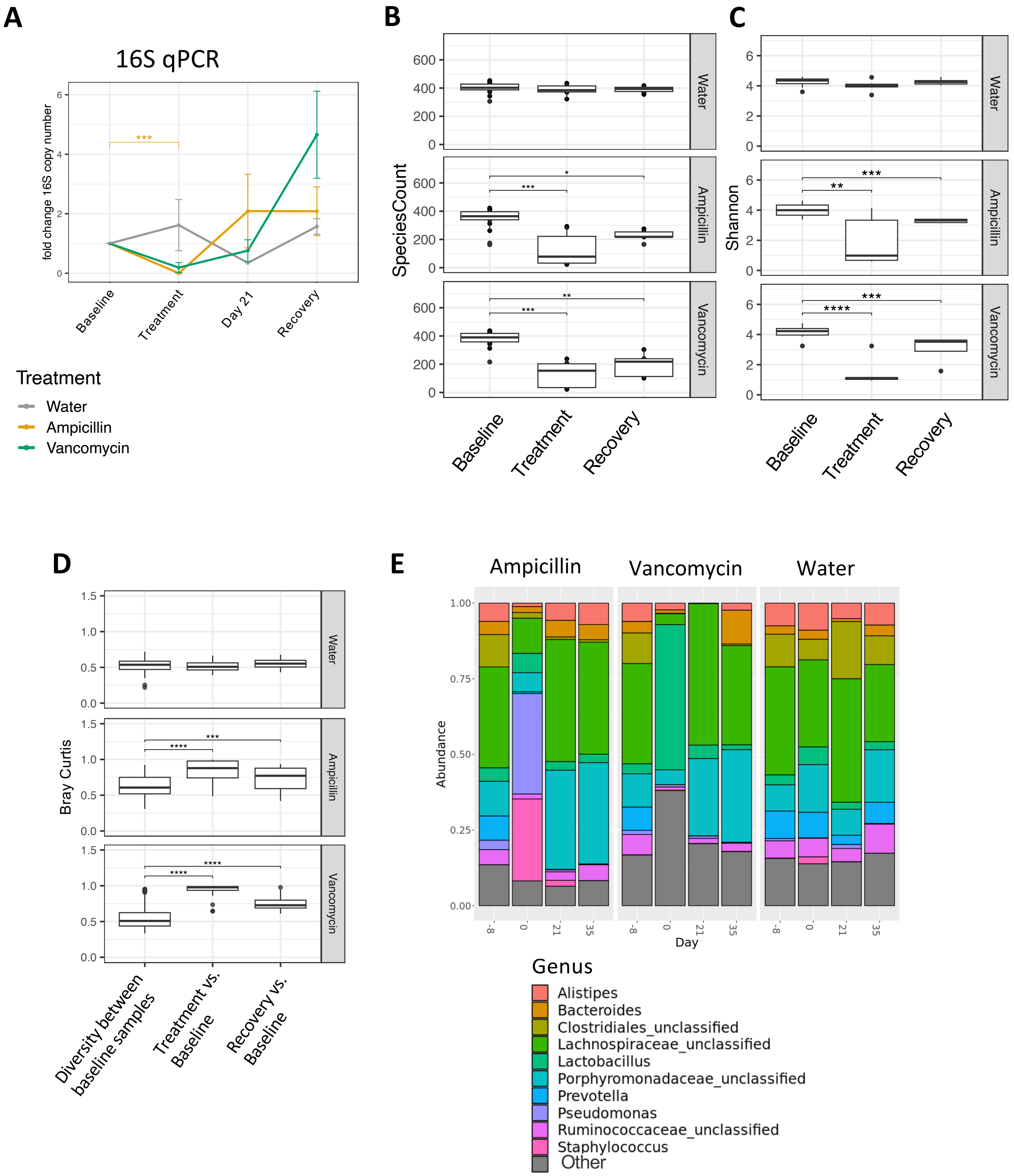
A
16S qPCR
fold change 16S copy number
***
Baseline
Treatment
Day 21
Recovery
Treatment
Water
Ampicillin
Vancomycin
B
SpeciesCount
Water
Ampicillin
Vancomycin
*
***
**
Baseline
Treatment
Recovery
C
Shannon
***
**
****
D
Bray Curtis
****
***
Diversity between baseline samples
Treatment vs. Baseline
Recovery vs. Baseline
E
Ampicillin
Vancomycin
Water
Abundance
Day
Genus
Alistipes
Bacteroides
Clostridiales_unclassified
Lachnospiraceae_unclassified
Lactobacillus
Porphyromonadaceae_unclassified
Prevotella
Pseudomonas
Ruminococcaceae_unclassified
Staphylococcus
Other

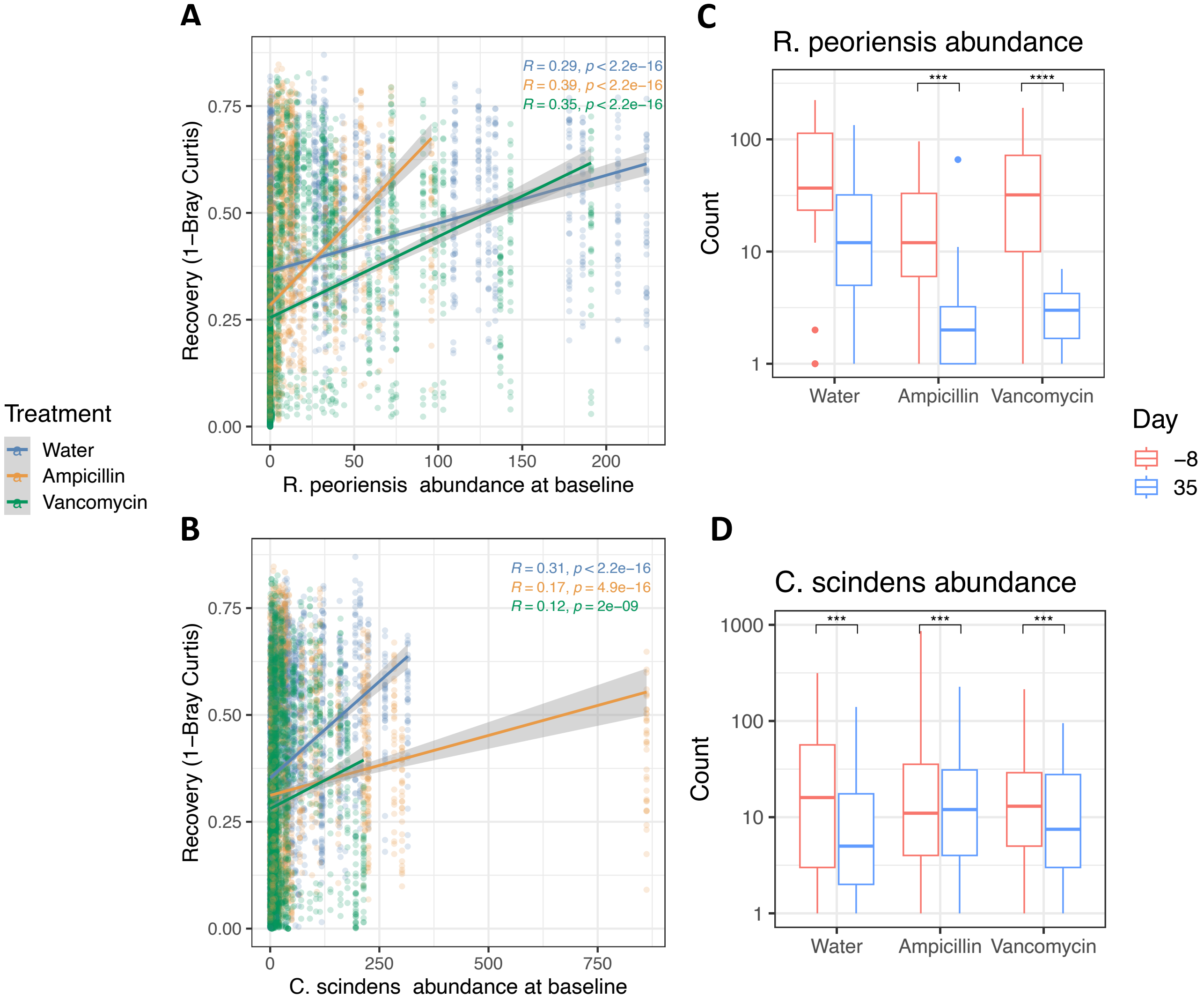

A
R = 0.29, p < 2.2e−16
R = 0.39, p < 2.2e−16
R = 0.35, p < 2.2e−16
Recovery (1−Bray Curtis)
R. peoriensis abundance at baseline
Treatment
Water
Ampicillin
Vancomycin
B
R = 0.31, p < 2.2e−16
R = 0.17, p = 4.9e−16
R = 0.12, p = 2e−09
Recovery (1−Bray Curtis)
C. scindens abundance at baseline
C
R. peoriensis abundance
Count
Water
Ampicillin
Vancomycin
Day
−8
35
D
C. scindens abundance
Count
Water
Ampicillin
Vancomycin
