# Supplementary material for: Spatial recovery of the murine gut microbiota after antibiotics perturbation
Source: mBio. 2024 Jun 4;15(7):e00707-24. doi: 10.1128/mbio.00707-24 (PMC11253616; doi:10.1128/mbio.00707-24)
Supplement: Supplemental Legends — Legends for supplemental tables and figures. [file mbio.00707-24-s0002.docx]

**Supplemental Figure Legends:**

**Supplemental Figure 1: Daily cage monitoring during antibiotic treatment. (A)** Estimated water intake per mouse **(B)** estimated food intake per mouse, (**C)** estimated dosage, per mouse (**D)** estimated daily change in bodyweight per mouse. Measurements were taken per cage (n = 3), and divided by number of mice in each cage (n = 2-6) leading to the estimated value per mouse. This estimated value was plotted per cage, representing the mean estimated value per mouse and error bars representing standard deviation.

**Supplemental Figure 2: 16S rRNA diversity analysis on mouse fecal samples. (A)** qPCR of the 16S rRNA gene at each timepoint for each treatment group. (**B)** Alpha diversity by species count and **(C)** Shannon diversity. **(D)** Beta diversity as measured by the Bray-Curtis dissimilarity index. **(D)** Taxonomic diversity at the genus level at each timepoint and treatment groups. The top 10 most abundant genera are plotted, with all other genera grouped as “Other”. Statistical analysis performed with the Kruskal–Wallis test with BH adjustment for multiple comparisons. Baseline n = 18, Treatment n = 12, Recovery n = 6. All comparisons were made to baseline.

**Supplemental Figure 3: Bacteria with high neighborhood diversity correlate with microbiome recovery. (A)** Recovery scores are plotted as 1 – Bray-Curtis dissimilarity index between baseline and recovery samples. The recovery score is plotted again the abundance of representative bacteria *R. peoriensis* at baseline **B)** Recovery score is plotted again the abundance of representative bacteria *C. scindens* at baseline. Pearson correlations and p-values are listed in each plot. **(C)** The abundance of *R. peoriensis* and **(D)** *C. scindens* at baseline and recovery. Statistical analysis performed with the Kruskal–Wallis test with BH adjustment for multiple comparisons made within tissue and treatment groups, n = 3.

**Supplemental Table Legends**

Supplemental Table 1: List of bacterial clusters identified by HiPR-FISH probes. Bacterial ID corresponds to the numbers used in Figure 5.

Supplemental Table 2: List of bacteria with significantly different neighborhoods in recovery after antibiotics treatment. Bacteria that have significantly different neighborhoods between treatment and the water-only control group in recovery are listed along with the variation explained (R^2^) and the bacteria with the largest coefficient. A positive coefficient is an increase in recovery and a negative coefficient is a decrease in recovery. Permanova was calculated using the adonis function in the vegan package, with 999 permutations for each tissue and treatment separately to get the full results on the effect of treatment (compared to the water-only control) in recovery (day 35).
